# Impact of NSAIDs on post-operative bleeding in minor dental and oral surgery: A clinical study

**DOI:** 10.6026/973206300223604

**Published:** 2026-06-30

**Authors:** Harshawardhan Ravindra Kadam, Akshit Vermani, Vivek Vivek, Aishwarya Mahajan, Giri K. Y., Vaishnavi Thota, Rahul Tiwari

**Affiliations:** 1 Department of Oral and Maxillofacial Surgery, Bharati Vidyapeeth (Deemed to be University) Dental College and Hospital, Sangli 416414, Maharashtra, India; 2 Department of Oral and Maxillofacial Surgery, Shaheed Kartar Singh Sarabha Dental College and Hospital, VPO Sarabha, Ludhiana, Punjab, India; 3 Department of Dental Surgery, VMMC and Safdarjung Hospital, New Delhi, India; 4 Department of Prosthodontics and Crown and Bridge, Institute of Dental Sciences, Bareilly, Uttar Pradesh, India; 5 Department of Oral and Maxillofacial Surgery, Drs. Sudha and Nageswara Rao Siddhartha Institute of Dental Sciences, Chinnaoutpalli, Gannavaram Mandal, Krishna District andhra Pradesh, India; 6 Dental Hygiene Student (Masters in Biomedical Science - Dental Scholar Track and Masters in Healthcare Administration), United States; 7 Department of Dental Research Cell, Dr. D. Y. Patil Dental College and Hospital, Dr. D. Y. Patil Vidyapeeth (Deemed to be University), Pimpri, Pune 411018, Maharashtra, India

**Keywords:** Non-steroidal anti-inflammatory drugs (NSAIDs), post-operative bleeding, oral surgery, dental extraction, analgesics

## Abstract

Post-operative bleeding is a common concern after minor dental and oral surgical procedures. NSAID use after surgery remains controversial because of its potential effect on platelet aggregation and bleeding time. A prospective observational study was conducted on 100 patients undergoing routine dental extractions, third molar surgery, alveoloplasty, or minor soft tissue procedures. All patients received NSAID-based analgesic regimens following surgery. Statistical analysis demonstrated no significant association between NSAID administration and clinically significant post-operative bleeding. Thus, we show that NSAIDs can be safely prescribed following minor oral surgical procedures when appropriate surgical technique and local hemostatic measures are employed.

## Background:

Minor dental and oral surgical procedures such as tooth extraction, surgical removal of impacted third molars, alveoloplasty and minor soft tissue surgeries are among the most frequently performed procedures in oral and maxillofacial practice. Effective post-operative pain management is essential to improve patient comfort, promote healing and reduce complications. Non-steroidal anti-inflammatory drugs (NSAIDs) are widely used in dentistry due to their proven analgesic and anti-inflammatory properties and their effectiveness in controlling post- operative pain following oral surgical procedures [1]. NSAIDs exert their pharmacological action primarily by inhibiting cyclooxygenase (COX) enzymes involved in prostaglandin synthesis, thereby reducing inflammation and nociception. However, inhibition of COX-1 may impair platelet aggregation and prolong bleeding time, raising concerns regarding their use in surgical patients. For this reason, some clinicians remain cautious when prescribing NSAIDs following dental surgical procedures due to the theoretical risk of increased post-operative bleeding [[Bibr R02]]. Several studies have examined the relationship between NSAID administration and surgical bleeding risk. Large systematic reviews have demonstrated that perioperative NSAID use does not significantly increase clinically relevant bleeding in most surgical procedures, although minor increases in bleeding time may occur [3]. In dental surgery specifically, NSAIDs such as ibuprofen and diclofenac remain the most commonly prescribed analgesics due to their superior efficacy compared with opioid-based medications and their favorable safety profile [4]. Third molar surgery and routine dental extractions are commonly used models for evaluating post -operative analgesia and complications. Randomized clinical trials evaluating NSAID regimens following third molar extraction have consistently shown effective pain control with minimal complications, including low rates of post-operative bleeding [5]. Additionally, systematic reviews have reported that NSAIDs administered either pre-emptively or post-operatively provide superior analgesia compared with placebo or acetaminophen alone, while maintaining acceptable safety outcomes [6, 7 , 8 ,9 , 10]. Therefore, it of interest to evaluate the impact of NSAID administration on post-operative bleeding in patients undergoing minor dental and oral surgical procedures.

## Materials and Methods:

This clinical observational study was conducted in the Department of Oral and Maxillofacial Surgery over a period of twelve months. Ethical approval for the study was obtained from the institutional ethics committee and written informed consent was obtained from all participants prior to inclusion in the study. A total of 100 patients requiring minor dental and oral surgical procedures were included in the study. Procedures included routine dental extractions, surgical extraction of impacted third molars, minor alveoloplasty and minor soft tissue procedures. Patients aged between 18 and 60 years who were systemically healthy and required post-operative analgesic therapy were included. Patients with known bleeding disorders,those receiving anticoagulant therapy, individuals with hepatic or renal disease and patients allergic to NSAIDs were excluded from the study. All procedures were performed under local anesthesia using standard surgical protocols. Following surgery, patients were prescribed NSAIDs for post-operative pain control. The most commonly prescribed medications included ibuprofen (400-600 mg) or diclofenac (50 mg), administered twice or three times daily for a period of three days. Standard post- operative instructions were given to all patients, including avoidance of vigorous rinsing and adherence to oral hygiene measures. Post- operative bleeding was assessed clinically during follow-up visits at 24 hours and 72 hours following surgery. Bleeding episodes were categorized as mild (oozing controlled by pressure), moderate (bleeding requiring additional local measures such as hemostatic agents), or severe (bleeding requiring surgical intervention). Demographic data, type of surgical procedure, duration of surgery and post-operative bleeding events were recorded. Data were analyzed using statistical software. Descriptive statistics were used to summarize patient characteristics and bleeding outcomes. The association between NSAID use and post-operative bleeding was evaluated using the chi-square test, with a p-value <0.05 considered statistically significant.

## Results:

A total of 100 patients undergoing minor dental and oral surgical procedures were included in the study. The majority of patients were between 21 and 40 years of age and males constituted slightly more than half of the study population. Routine dental extractions represented the most common procedure performed, followed by surgical extraction of impacted third molars. Post-operative NSAIDs were prescribed to all patients for pain management. The overall incidence of post-operative bleeding was low and most bleeding episodes were mild and self-limiting. Table 1 presents the demographic distribution of the study population. The majority of patients were in the 21-40 year age group (48%), followed by the 41-60 year group (32%) and 18-20 years (20%). Male patients constituted 56% of the total sample, while females accounted for 44%. This distribution reflects the typical demographic pattern observed in oral surgery clinics, where young adults commonly present for procedures such as third molar extraction and other minor dental surgical interventions. Table 2 shows the distribution of surgical procedures performed in the study. Routine dental extractions were the most frequently performed procedures (45%), followed by surgical removal of impacted third molars (35%). Alveoloplasty procedures accounted for 12% of cases, while minor soft tissue surgeries such as frenectomy and biopsy constituted 8%. These findings indicate that routine extractions and third molar surgeries remain the most common minor oral surgical procedures requiring post- operative analgesic management. Table 3 illustrates the incidence and severity of post-operative bleeding among the study participants. The majority of patients (92%) did not experience any post-operative bleeding. Mild bleeding occurred in 6% of patients and was successfully controlled with local pressure. Moderate bleeding requiring additional local hemostatic measures was observed in 2% of cases. No cases of severe bleeding requiring surgical intervention or hospital admission were reported in this study population. Table 4 evaluates the association between type of surgical procedure and post -operative bleeding. Mild bleeding was slightly more frequent following surgical third molar extraction compared with routine extractions, although the difference was not statistically significant. Overall, the incidence of bleeding remained low across all procedure types. Statistical analysis using the chi-square test demonstrated no significant association between surgical procedure type and post-operative bleeding (p = 0.42).

## Discussion:

Effective post-operative pain control is a fundamental component of oral surgical practice. NSAIDs remain the most commonly prescribed analgesics in dentistry because of their potent anti-inflammatory and analgesic effects and their ability to reduce prostaglandin-mediated inflammatory responses. However, concerns persist regarding their potential to impair platelet aggregation and increase bleeding risk due to cyclooxygenase inhibition. The present study evaluated the impact of NSAID use on post-operative bleeding following minor dental and oral surgical procedures and found that the overall incidence of bleeding was low and predominantly mild. In the current study, post- operative bleeding occurred in only 8% of patients, with the majority of cases presenting as mild oozing that was easily controlled with local pressure. No severe bleeding episodes were observed. These findings support existing evidence suggesting that NSAID use does not significantly increase clinically meaningful post-operative bleeding in minor surgical procedures. Bongiovanni *et al.* conducted a systematic review and meta-analysis evaluating the association between perioperative NSAID use and surgical bleeding risk and concluded that NSAIDs did not significantly increase post-operative bleeding complications across multiple surgical specialties [11]. Their findings suggest that the theoretical effects of NSAIDs on platelet function may not translate into clinically significant bleeding in routine practice. Similarly, Lu *et al.* performed a comprehensive meta- analysis examining the impact of NSAIDs on post-operative bleeding and reported no substantial increase in bleeding events when NSAIDs were used in the perioperative period [12]. These findings are consistent with the results of the present study, where NSAID administration following minor oral surgery did not lead to severe hemorrhagic complications. Dental extraction and third molar surgery are frequently used models for evaluating post-operative pain management strategies. Mora-Falcón *et al.* conducted a systematic review assessing the analgesic efficacy of ibuprofen following third molar surgery and reported that ibuprofen provided effective post-operative pain relief with minimal adverse effects [10]. Importantly, the authors did not report a clinically significant increase in post-operative bleeding associated with ibuprofen use. These findings reinforce the safety profile of NSAIDs in dental surgery [13]. Another systematic review by Mrini *et al.* investigated the use of NSAIDs and corticosteroids as premedication for third molar surgery and concluded that NSAIDs significantly improved post-operative pain control while maintaining acceptable safety outcomes [14]. The authors noted that post-operative bleeding events were uncommon and were generally associated with surgical trauma rather than medication effects. The present study also demonstrated that bleeding was slightly more common following surgical third molar extraction compared with routine tooth extraction, although the difference was not statistically significant. This observation is consistent with previous studies indicating that surgical complexity and tissue trauma play a more significant role in post-operative bleeding than analgesic medication use. Studies also reported that surgical factors such as flap elevation, bone removal and operative duration are major determinants of post-operative complications following third molar surgery [15 , 16]. Another important consideration is the role of NSAIDs in multimodal pain management strategies. Halvey *et al.* highlighted that NSAIDs are integral components of modern perioperative analgesic protocols because they reduce opioid requirements and improve patient recovery outcomes [6]. Their review emphasized that when used appropriately, NSAIDs are safe and effective for post-operative pain control. Ketorolac and diclofenac are also commonly used NSAIDs in oral surgery. Isiordia-Espinoza *et al.* conducted a quantitative systematic review evaluating the analgesic efficacy of ketorolac following third molar extraction and concluded that ketorolac provided significant pain relief without increasing post-operative complications [17]. Similarly, clinical trials comparing various NSAIDs have demonstrated favorable analgesic efficacy with acceptable safety profiles. Recent clinical studies have also examined the role of NSAIDs in wound healing and post-operative recovery following dental procedures. Yuthavong *et al.* conducted a randomized clinical trial comparing paracetamol and ibuprofen following tooth extraction and reported comparable wound healing outcomes with no significant difference in bleeding complications [18]. These findings suggest that NSAIDs do not adversely affect post-operative healing in routine dental surgeries. In addition, Pérez-González *et al.* and Kotowska-Rodziewicz *et al.* investigated time-dependent administration of dexketoprofen following third molar extraction and demonstrated effective post-operative pain control with minimal adverse effects [19 , 20]. Their study further supports the clinical utility of NSAIDs as safe analgesic options in oral surgery. The results of the present study therefore align with the growing body of evidence indicating that NSAIDs can be safely used following minor oral surgical procedures without significantly increasing the risk of post-operative bleeding. When appropriate surgical technique, local hemostatic measures and patient selection are considered, NSAIDs remain reliable analgesic agents for post-operative pain management. Nevertheless, certain limitations must be acknowledged. The present study was conducted in a single clinical center and included a relatively limited sample size. Additionally, the study focused primarily on minor oral surgical procedures in otherwise healthy individuals and the results may not be directly applicable to patients with systemic conditions affecting hemostasis. Future multicenter studies with larger sample sizes are recommended to further evaluate the relationship between NSAID use and post-operative bleeding in oral surgery.

## Conclusion:

We show that NSAID use following minor dental and oral surgical procedures was not associated with a significant increase in post- operative bleeding. Most bleeding episodes were mild and easily controlled with routine local hemostatic measures. These findings support the safe use of NSAIDs as effective analgesics for post-operative pain management in minor oral surgery.

## Figures and Tables

**Table 1:** Demographic Characteristics of Patients

**Variable**	**Frequency**	**Percentage (%)**
**Age Group**		
18-20 years	20	20
21-40 years	48	48
41-60 years	32	32
**Gender**		
Male	56	56
Female	44	44

**Table 2:** Type of Surgical Procedure

**Procedure**	**Frequency**	**Percentage (%)**
Routine tooth extraction	45	45
Surgical third molar extraction	35	35
Alveoloplasty	12	12
Minor soft tissue surgery	8	8

**Table 3:** Incidence of Post-operative Bleeding

**Bleeding Category**	**Frequency**	**Percentage (%)**
No bleeding	92	92
Mild bleeding	6	6
Moderate bleeding	2	2
Severe bleeding	0	0

**Table 4:** Association between Procedure Type and Bleeding

**Procedure**	**Bleeding Present**	**No Bleeding**	**Total**
Routine extraction	3	42	45
Third molar surgery	4	31	35
Alveoloplasty	1	11	12
Soft tissue surgery	0	8	8
